# Cloning and characterization of the lignin biosynthesis genes *NcCSE* and *NcHCT* from *Neolamarckia cadamba*

**DOI:** 10.1186/s13568-019-0860-z

**Published:** 2019-09-21

**Authors:** Juncheng Li, Xiaoling Huang, Hao Huang, Heqiang Huo, Chi D. Nguyen, Ruiqi Pian, Huaqiang Li, Kunxi Ouyang, Xiaoyang Chen

**Affiliations:** 1 Guangdong Key Laboratory for Innovative Development and Utilization of ForestPlant Germplasm, South China Agricultural University, Wushan Road 483, Tianhe District, Guangzhou, 510642 China; 2 Guangdong Key Laboratory of Tropical and Subtropical Fruit Tree Research, Institute of Fruit Tree Research, Guangdong Academy of Agricultural Sciences, Guangzhou, 510640 China; 3 Guangdong Province Research Center of Woody Forage Engineering Technology, College of Forestry and Landscape Architecture, South China Agricultural University, Guangzhou, 510642 China; 4 State Key Laboratory for Conservation and Utilization of Subtropical Agro-bioresources, Guangzhou, China; 5 Guangxi Botanical Garden of Medical Plants, Nanning, 530023 China; 6 Mid-Florida Research and Education Center, Institute of Food and Agricultural Sciences, University of Florida, Apopka, FL USA; 7 State_run Leizhou Forestry Bureau, Zhanjiang, 524348 Guangdong China

**Keywords:** Lignin, *NcCSE*, *NcHCT*, *Neolamarckia cadamba*, DEGs

## Abstract

*Neolamarckia cadamba* is an important fast growing tree species used for pulping and wood material in industry for it’s desirable wood properties. As one of the most important content in wood, lignin provides structural integrity, strength, and hydrophobicity to the thickened cell walls and is the major factor contributing to biomass recalcitrance. It does not reduce the palatability of forage grass for animals, but it hinders the isolation of cellulose fibers and the efficient enzymatic depolymerization of cellulose and hemicellulose into fermentable sugars in biorefining processes by limiting the access by hydrolytic enzymes to their polysaccharide substrates. This work focused on analyzing the functions of *NcCSE* (Caffeoyl Shikimate Esterase, GenBank accession number: MG739672) and *NcH*CT (Hydroxycinnamoyl Transferase,GenBank accession number: MG739673) in the lignin biosynthetic process in order to improve the potential for utilization of leaves and wood from *N. cadamba*. The mutant phenotype of *cse*-*2* was dramatically complemented to WT in the stable transgenic lines *cse*-*35S::NcCSE*, but overexpression of *NcHCT* in the *cse*-*2* mutant did not have the same result as *cse*-*35S::NcCSE*, providing only partial complementation.

## Introduction

*Neolamarckia cadamba* (Rubiaceae*, Anthocephalus*) is a tropical evergreen tree species with a natively distribution in South and Southeast Asia. It is famous for its fast growing and perfect trunk, which can reach a height of 45 m with a DBH (diameter at breast height) of 100–160 cm (Krisnawati et al. 2011). It is excellent for use in building, furniture fabrication and papermaking. Moreover, it is characterized by an abundance of alkaloids in the leaves and bark, such as cadambine and dihydroconchonine, which can be used as treatments for several diseases like fever, anemia, diabetes, tumors, and have been studied for more than 100 years (Dwevedi et al. 2015). In our previous research, we found that the leaves of *N. cadamba* contained an abundance of protein and fat, and the compositional parameter index as a forage is even better than that of the common forage species Alfalfa (*Medicago sativa* Linn) (Additional file 1: Table S1), so it is also good as a woody forage plant.

The plant cell wall, which is the most abundant renewable energy in nature, the main component of plant biomass and essential mechanical support for the plant, is a complex matrix of several different polymers enclosing the plasma membrane, maintaining cell shape and protecting the cell (Hückelhoven 2007). The major components making up the framework of the cell wall are cellulose organized into microfibrils, hemicelluloses and lignin filling the space between the microfibrils (Salmén 2015; Adel et al. 2016). Of these compounds, lignin is the major factor limiting the digestibility of forage dry matter, the conversion of lignocellulosic biomass to fermentable sugars, and the effectiveness of pulping in papermaking (Powell et al. 2017; Zeng et al. 2014; Constant et al. 2016).

Lignin is a complex polymer made from different kinds of monomeric subunits linked via ether bonds or carbon–carbon bonds, and the most abundant of these units are guaiacyl (G), syringyl (S), and *p*-hydroxyphenyl (H) groups (Shuai et al. 2016). Lignin is synthesized via shikimate through the general phenylpropanoid pathway and a monolignol-specific pathway, which include 10 well-known enzymes (PAL, phenylalanine ammonia-lyase; C4H, cinnamate 4-hydroxylase; 4CL, 4-coumarate: CoA ligase; HCT, p-hydroxycinnamoyl-CoA: quinate shikimate p-hydroxycinnamoyltransferase; C3H, *p*-coumarate 3-hydroxylase; CCoAOMT, caffeoyl-CoA O-methyltransferase; CCR, cinnamoyl-CoA reductase; F5H, ferulate 5-hydroxylase; COMT, caffeic acid O-methyltransferase; CAD, cinnamyl alcohol dehydrogenase) (Boerjan et al. 2003; Vanholme et al. 2010; Ouyang et al. 2016), and a more recently identified enzyme, CSE (Caffeoyl shikimate esterase), which also participates in this process (Vanholme et al. 2013). Many studies have identified the role of the first 10 enzymes in lignin biosynthesis process, and the model of lignin biosynthesis based on these 10 enzymes has been accepted for more than 10 years. However, for the new member, more work needs to be done in order to verify its position in lignin biosynthesis. It is believed that CSE and HCT may have a similar function in this biosynthetic process, as both of them can hydrolyze caffeoyl shikimate, a well-known intermediate in lignin biosynthesis, to produce caffeic acid or caffeoyl-CoA (Vanholme et al. 2013). In Saleme’s study, down-regulation of *CSE* in hybrid poplar result in a reduced lignin deposition and relatively higher cellulose content (Saleme et al. 2017). But, the relation of *CSE* and *HCT* is not mentioned. In this study, we are trying to determine the functions and relationship of *NcCSE* and *NcHCT* in the process of lignin biosynthesis and there potential influence in biomass accumulation, cell wall deposition, lignin per percentage and composition by heterologous express the *NcCSE* and *NcHCT* in *Arabidopsis cse*-*2* mutant and wild type plant. We find that NcCSE can fully complement ate the cse-2 while NcHCT can only providing partial complementation.

## Materials and methods

### Plant materials and growth conditions

*Neolamarckia cadamba* plants were cultivated in an open field in 5-l pots for 2 years, and *A. thaliana* was cultivated in a growth chamber at a light intensity of 150 µM m^2^/s under a 16 h light/8 h dark photoperiod and a temperature setting of 22 ± 1 °C light/20 ± 1 °C dark with 80% humidity. All the plants were generated from seed. *N. cadamba* seeds were collected from Guangzhou, China, and *A. thaliana* mutant seeds were ordered from ABRC. In this study, the *cse*-*2* (SALK_023077) T-DNA insertion mutant and Col-0 wild type *Arabidopsis* were used. The insertion locus of the *cse*-*2* is shown in Additional file 1: Fig. S1.

### Gene cloning and vector construction

According to our previous RNA-sequence analysis, based on the *A. thaliana AtCSE* (AT1G52760) sequence, *NcCSE* and *NcHCT* were identified from our RNA-seq database (NCBI BioProject Accession: PRJNA232616) through blastx homology searching (The e-value cut-off is 1e-10, those with the highest homology were chose). These two genes were amplified by nested PCR, firstly using the gene specific primer pair N1, then the PCR product was used as the template for amplification with the gene specific primer pair N2 which contained the attB1 and attB2 adapter at the 5′ end for *NcCSE* and *NcHCT* respectively. All primers used in this study are listed in Additional file 1: Table S2. The product was subcloned with a BP reaction (Gateway cloning) into the pDONR221 vector after purification. Sequence identity was confirmed by sequencing and the fragment was subsequently introduced into the pEarley100 vector by means of LR Clonase (Invitrogen, USA). In the final construct, expression of the gene was driven by a CaMV 35S promoter with a basta-resistance (bar) gene as plant selection marker. Two vectors, *35S::NcCSE* and *35S::NcHCT,* were constructed in this study and transferred into *Agrobacterium tumefaciens* strain GV3101.

### Generation of transgenic *Arabidopsis*

The floral dip method was used for *cse* mutant transformation (Zhang et al. 2006). Two different transgenic plant lines, *cse*-*35S::NcCSE* and *cse*-*35S::NcHCT*, were generated and the seeds of each transformed T_0_ line were harvested after maturation. The herbicide basta (glufosinate ammonium) was used for screening the positive transgenic plants. For each transformation, three independent homozygous transgenic lines were used for experimentation, after selfing for two generations and genotyping.

### Co-expression analysis of lignin biosynthesis genes

To investigate the expression profile of genes related to lignin biosynthesis, especially genes related to *AtCSE*, in both wild type and transgenic plants, co-expression analysis basedon the Topology Overlap Matrix (WGCNA) was used to identify those genes. Microarray data downloaded from GEO (https://www.ncbi.nlm.nih.gov/geo/) consisted of 53 stem samples in 7 datasets, GSE53580 (GSM1296372-GSM1296383), GSE5633 (GSM131643-GSM131645, GSM131655-GSM131660), GSE6151 (GSM142623-GSM142625, GSM142629-GSM142631, GSM142635-GSM142637), GSE23801 (GSM587036, GSM587037), GSE24763 (GSM609886-GSM609891), GSE24781 (GSM610374-GSM610385), GSE2848 (GSM62697, GSM62701, GSM62705), and the raw data were normalized with the Robust Multichip Average (RMA) normalization protocol. The fifteen genes showing the strongest relationship with *AtCSE*, five lignin biosynthesis genes sharing co-expression with *AtCSE*, and six cellulose biosynthesis genes that also showed strong correlation with *AtCSE* were selected for qRT-PCR analysis.

### Phenotype analysis of T3 generation transgenic plants

Hypocotyl length was measured by ImageJ (https://imagej.net/) on the 7th day and 14th day after planting under the culture conditions described above; leaf area was measured by ImageJ on the 21st day, with the mean area of the 4th, 5th and 6th true leaves representing the leaf area;, and at the same time the number of rosette leaves was counted for each plant. The rosette leaf biomass was measured on the 35th day after oven drying at 40 °C, and the plant height and dry above ground biomass were measured on the 50th day. The stem thickness was expressed as the cross sectional area of the main stem at 3–4 mm above ground level on the 50th day, which was embedded in 3% (W/V) agar and cut using a LeicaVT1000S vibratome equipped with a razor blade. The stem sections were observed under an Olympus BX43F light microscope and measured with ImageJ.

### RNA extraction and quantitative Real-Time PCR (qRT-PCR)

*Neolamarckia cadamba* total RNA was extracted with a protocol using CTAB plus an OMEGA Plant RNA Isolation Kit (Ouyang et al. 2014); *A. thaliana* total RNA was extracted with an OMEGA Plant RNA Isolation Kit alone (Omega Bio-Tek, Doraville, CA) following the manufacturer’s instructions. First-strand cDNA was synthesized from 1 μg RNA with Prime Script™ RT Master Mix (Takara, Japan) according to the manufacturer’s instructions. The cDNA was diluted with a 14-fold volume of ddH_2_O for use as the template in qRT-PCR. SYBR Premix Ex Taq™ Kit (Takara, Japan) was used in the qRT-PCR, and the amplification conditions were as follows: initial denaturation at 95 °C for 30 s, amplification and quantification at 95 °C for 5 s, 58 °C for 30 s, and 72 °C for 15 s for 40 cycles (with a single fluorescence measurement), and an indefinite hold at 10 °C. After the final PCR cycle, a melting curve program was performed to determine the specificity of the PCR products by heating from 65 to 95 °C with a heating rate of 0.1 °C/s and continuous fluorescence measurement. The primers used for qRT-PCR are listed in Additional file 1: Table S3; the *N. cadamba* gene *Cyclophilin* (JX902587) and the *A. thaliana* gene *PP2AA3* (AT1G13320) were included as internal references. The relative levels of expression of the two genes *NcCSE* and *NcHCT* in the basal, middle and apical stem segments of *N. cadamba* were analyzed by qRT-PCR. All of the 26 genes selected by co-expression analysis in *A. thaliana* were analyzed by qRT-PCR in the basal stem (1st and 2st internode) on the 7th day after bolting and in rosette leaves on the 21st day after germination.

### Microscopic evaluation and visualizing of lignin content by histochemical staining

The stem of 1 cm above the cotyledonary node were embedded and cut using a LeicaVT1000S vibratome as described above or fixed with FAA (formaldehyde:glacial acetic acid:70% ethanol [1:1:18]), embedded in paraffin and cut with a rotary slicer (RM 2235, LEICA, Germany). Safranin and fast green staining was used to differentiate lignified and non-lignified tissues (Mason 1961). Lignin and cellulose are stained from red to pink and blue to green respectively, depending on their content, and the lignin content can be roughly determined after staining.

### Cell wall characterization and lignin composition

Determination of lignin content was based on the protocol of Ververis with minor modifications (Ververis et al. 2007). The samples were dried at 40 °C, and ground to a fine powder. Then each sample (1.000 ± 0.050 g powder; *W1*) was incubated in 10 ml 72% (v/v) H_2_SO_4_ solution at 100 °C for 5 h to degrade the cellulose and hemi-cellulose, and filtered through a sintered glass crucible. The solid residue (*W2*) obtained after drying at 105 °C for 24 h was heated at 600 °C for 5 h and the weight of the residue (*W3*) after cooling down was measured. The acid insoluble lignin content (%) is given by (*W2*–*W3*)/*W1.*

To calculate the weight of the cell wall residue (CWR), about 2 g (*W4*) of the powder described above was extracted sequentially with water, ethanol, chloroform, and acetone to obtain the residue (weight *W5*) after drying at 40 °C, and CWR (%) was calculated as *W5*/*W4*. The content of cellulose is determined totally follow the method descript by Ouyang (Ouyang et al. 2013).

Wiesner’s staining combined with Maule’s reagent was used for roughly determining the S/G lignin composition ratio (Lewis and Yamamoto 1990; Guo et al. 2001; Trabucco et al. 2013). However, lignin monomer composition was more accurately quantified by Gas Chromatography (HP 5890 series II Plus GC Systems) according to a protocol for analysis of DFRC monomers (20 mg stem CWR samples for each measurement; with 0.05 mg tetracosane as internal standard) (Lu and Ralph 1997). All the measurements above were carried out in triplicate.

## Results

### Lignin content and model of gene expression in *N. cadamba*

Both *NcCSE* and *NcHCT* had a higher expression level in the basal and middle segments than in the top segment of the *N. cadamba* stem, which was consistent with the trend of lignin content in these three segments (Table 1).

**Table 1:** Expression levels of *NcCSE* and *NcHCT*, and lignin and cellulose contents, in apical, middle and basal stem segments

Segment	*NcHCT*	*NcCSE*	Lignin (%)	Cellulose (%)
Apical	0.11 (0.03)	1.09 (0.17)	12.81 (1.33)	49.62 (3.45)
Middle	0.42 (0.12)***	4.74 (0.83)***	25.47 (2.75)***	39.93 (3.84)**
Basal	0.78 (0.21)***^†††^	4.25 (0.69)***	23.19 (3.37)***	42.55 (1.99)*

Apical, middle and basal indicate apical, middle and basal 10 cm segments of the stem. The data outside and in the brackets are mean value and SEM (n = 3), respectively

*, ^†^ Indicate significant differences (0.01 < p < 0.05) compared with, respectively, the apical and middle stem segments (** and ^††^ 0.001 < p<0.01), and (*** and ^†††^ p < 0.001)

### Transgenic plant lines generated

The seedlings were screen with basta, six (*cse*-*35S::NcCSE1, cse*-*35S::NcCSE2, cse*-*35S::NcCSE3, cse*-*35S::NcHCT1, cse*-*35S::NcHCT2*, *cse*-*35S::NcHCT3* six overexpression lines *WT*-*35S::NcCSE1, WT*-*35S::NcCSE2, WT*-*35S::NcCSE3, WT*-*35S::NcHCT1, WT-35S::NcHCT2* and *WT*-*35S::NcHCT3*) of the complementary transgenic lines generated were randomly choosed for phenotyping, the presence of the insertion was confirmed by PCR with gene specific primers, all of the six lines were single-copy insertion (verified by Chi square test, Additional file 1: Tables S4, S5). T3 generation transgenic plants were used for the following analysis.

### Co-expression analysis of *CSE* and lignin biosynthesis related genes and qRT-PCR

**Fig. 1:**
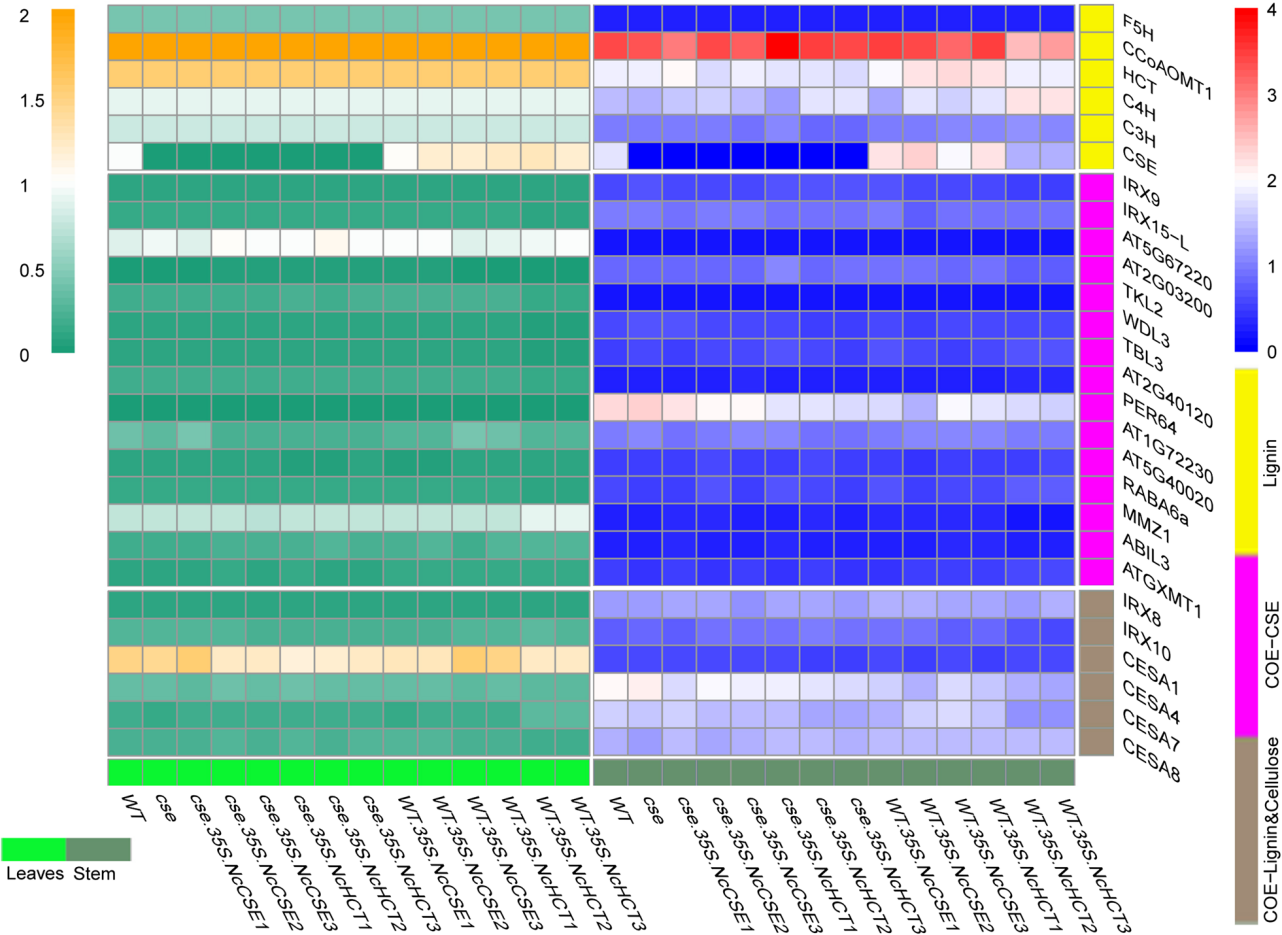
Relative expression level of some *A. thaliana* genes relevant to the lignocellulosic biosynthesis process in different lines and tissues

**Fig. 2:**
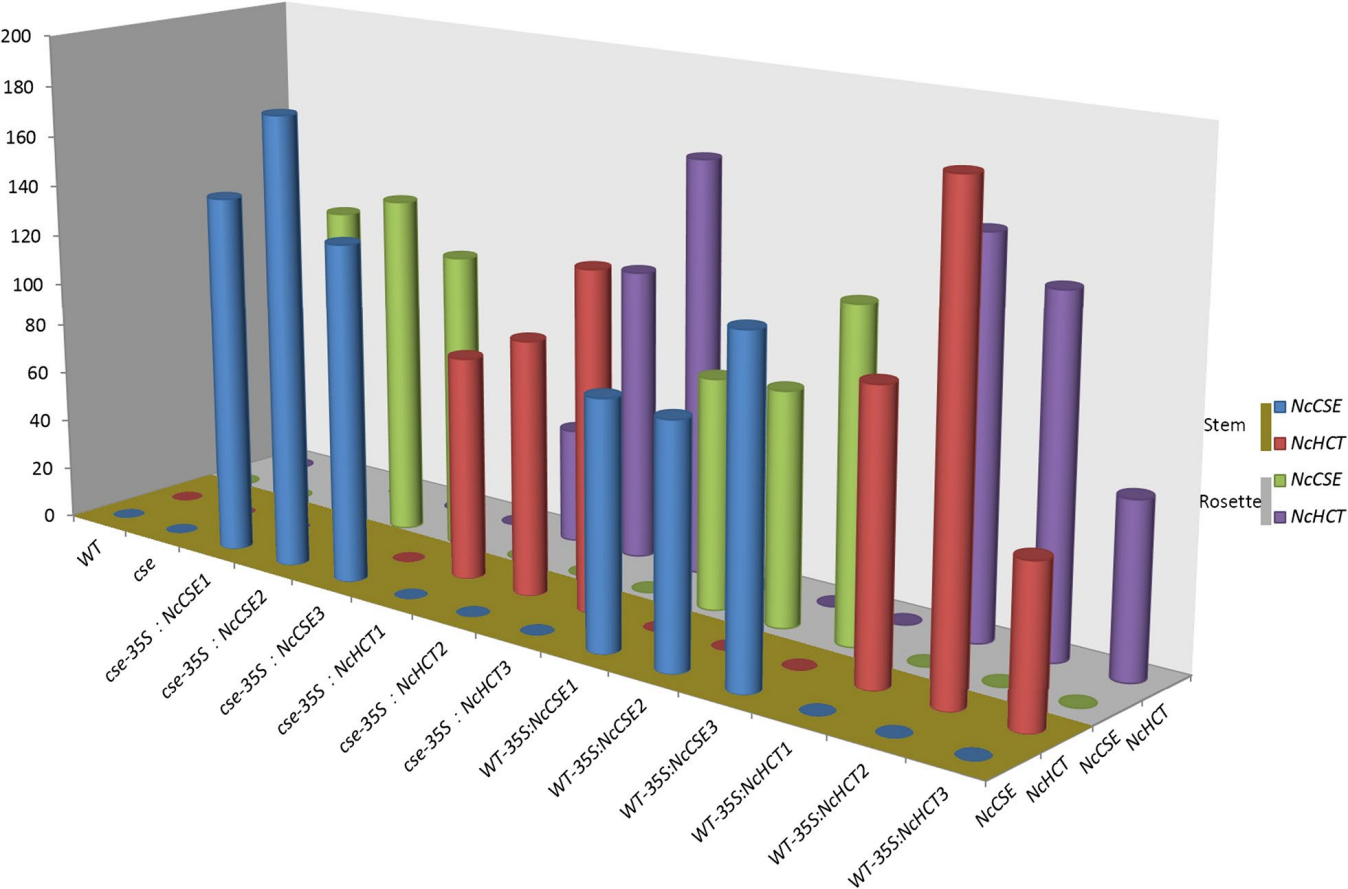
Relative expression levels of the exogenous genes *NcCSE* and *NcHCT* in transgenic lines and in wild-type and *cse* mutant plants

In the results of co-expression analysis of all genes in the lignin biosynthesis pathway, four of them, *F5H*, *C3H*, *C4H* and *CCoAOMT1* shared many more co-expressed genes with *CSE* compared with the other lignin biosynthesis pathway genes (Additional file 1: Figs. S2, S3). The 15 genes having the strongest relationship with *CSE*, and the other six genes that correlated with *CSE* in the cellulose biosynthesis pathway (Additional file 1: Table S6), were selected for analysis with qRT-PCR. However, many of the genes above showed no significant difference between transgenic lines and WT or the *cse* mutant in either stem or rosette leaves, the exceptions were *C3H*, *C4H*, *HCT* and *CCoAOMT1*, which had lower expression levels in the *cse* mutant compared with WT and *cse*-*35S::NcCSE* lines (Fig. 1), suggesting that genes other than these four were not influenced by the gene overexpression or the *cse* mutation and would not be likely to influence the phenotypes of those lines. In transgenic *NcCSE* and *NcHCT* overexpression plants, the overexpressed genes had a very high expression level in every line (Fig. 2).

### Hypocotyl and inflorescence stem analysis

**Fig. 3:**
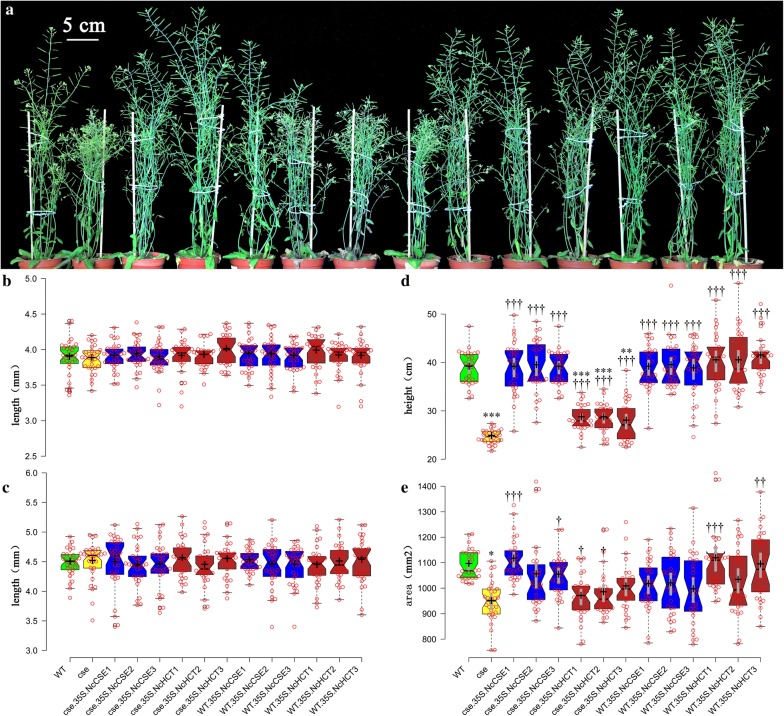
Phenotype statistics for the 14 lines, Thirty plants were measured for each line, **a** representative plants of each line; **b** hypocotyl lengths at day 7 after planting; **c** hypocotyl lengths at day 14 after planting; **d** plant height at day 50 after planting; **e** main stem diameter at day 50 after planting

**Fig. 4:**
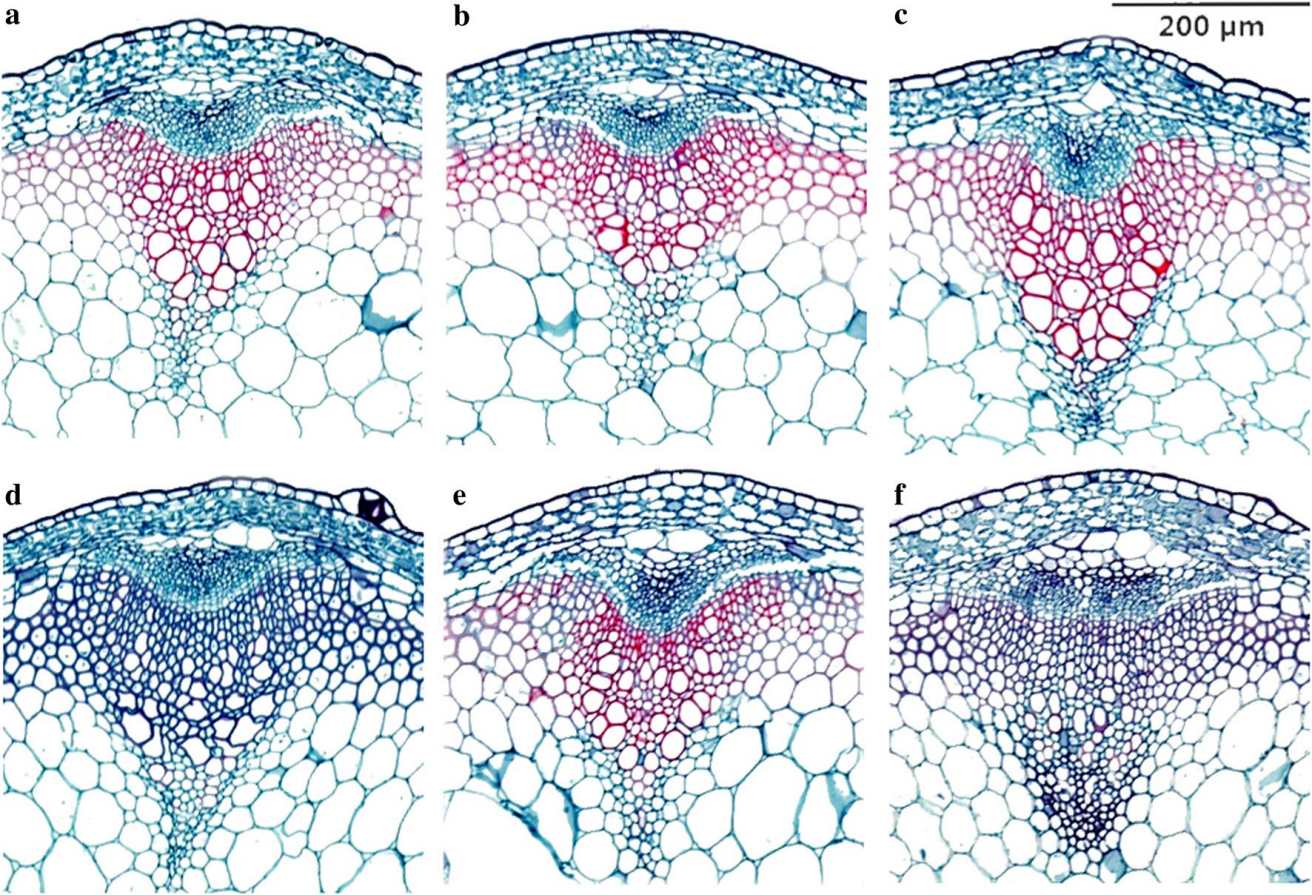
Paraffin sections of stems of representative lines stained with safranin and fast green. **a** WT; **b** *WT*-*35S:NcCSE*; **c** *WT*-*35S:NcHCT*; **d** *cse*; **e** *cse*-*35S:NcCSE*; **f** c*se*-*35S:NcHCT*; bar = 200HCT

The hypocotyl length in seedlings by the 7th day and 14th day showed no significant difference between transgenic plants and WT or *cse* mutant plants (Figs. 3b, 4c, Additional file 1: Table S7), but the *cse* mutant was significantly shorter, by 34.3%, than the WT by day 50. However, the height of *NcCSE* complemented *cse* lines was restored to that of WT plants. Although the *NcHCT* complemented *cse* lines also significantly increased (by 11.4%, 11.4%, 8.7%, p < 0.01) in height, they did not reach the WT height. The similarity in hypocotyl length during early growth of the plants and the difference between the height of the plants at day 50 (Fig. 3a, d, Additional file 1: Table S7) suggested that *CSE* does not affect stem tissue development during the period of primary growth (Sibout et al. 2008), but after bolting, when secondary growth and deposition of lignin occur, differences in the lignification process mediate the different heights of these lines. The stem cross-section area of the *cse* mutant was a little smaller (4.9%, p = 0.049) than that of WT, but it was restored by complementation in all the transgenic lines (Fig. 3e, Additional file 1: Table S7).

### Biomass comparison

Biomass is another index used to evaluate plant development. None of the lines had any significant difference in the dry weight of rosette leaves, though the dry weight varied among the different lines (Additional file 1: Table S8). However, the dry weight of the main inflorescence stem of the *cse* mutant was significantly less than that of WT. *NcCSE* overexpression could restore it to the WT level in *cse*-*35S::NcCSE* lines, and *cse*-*35S::NcHCT* lines were significantly heavier than the *cse* mutant but significantly lighter than WT. In addition, the dry weight of the total aboveground part of the *cse* mutant was less than those of the other lines at day 50, but there was no significant difference between WT and the transgenic lines (Additional file 1: Table S8). At the 21st day after planting, the area and number of leaves of the *cse* mutant were similar to those of WT and the transgenic lines (Additional file 1: Table S8). The dry weight, and number and area of leaves, in the *cse* mutant were not significantly different compared with WT and the transgenic lines, suggesting that leaf development is not severely affected by the *cse* mutation although the growth and development of the main inflorescence stem are severely inhibited by this mutation. Furthermore, the transgenic lines showed complete or partial restoration of the height and dry weight of the main inflorescence stem, so that the final dry weight of the total aboveground part of the *cse* mutant was less than in the other lines.

### Cell wall residue comparison

Cell wall residue (CWR) extraction is a good way to explore the composition of plant dry weight. The CWR of the dry weight of *cse* mutant rosette leaves and main inflorescence stem was reduced by, respectively, about 14.3% and 10.5% compared with WT. The overexpression of *NcCSE* in the three independent *cse*-*35S::NcCSE* lines restored the CWR of the main inflorescence stem to the WT level in all cases (Additional file 1: Table S8). However, two of the three *cse*-*35S::NcCSE* lines still had significantly less CWR than WT in the rosette leaves, but all of them had significantly more CWR than the *cse* mutant. There was no significant difference between *cse*-*35S::NcHCT* and the *cse* mutant with respect to the CWR of either the main inflorescence stem or the rosette leaves (Additional file 1: Table S8).

### Histochemical staining

**Fig. 5:**
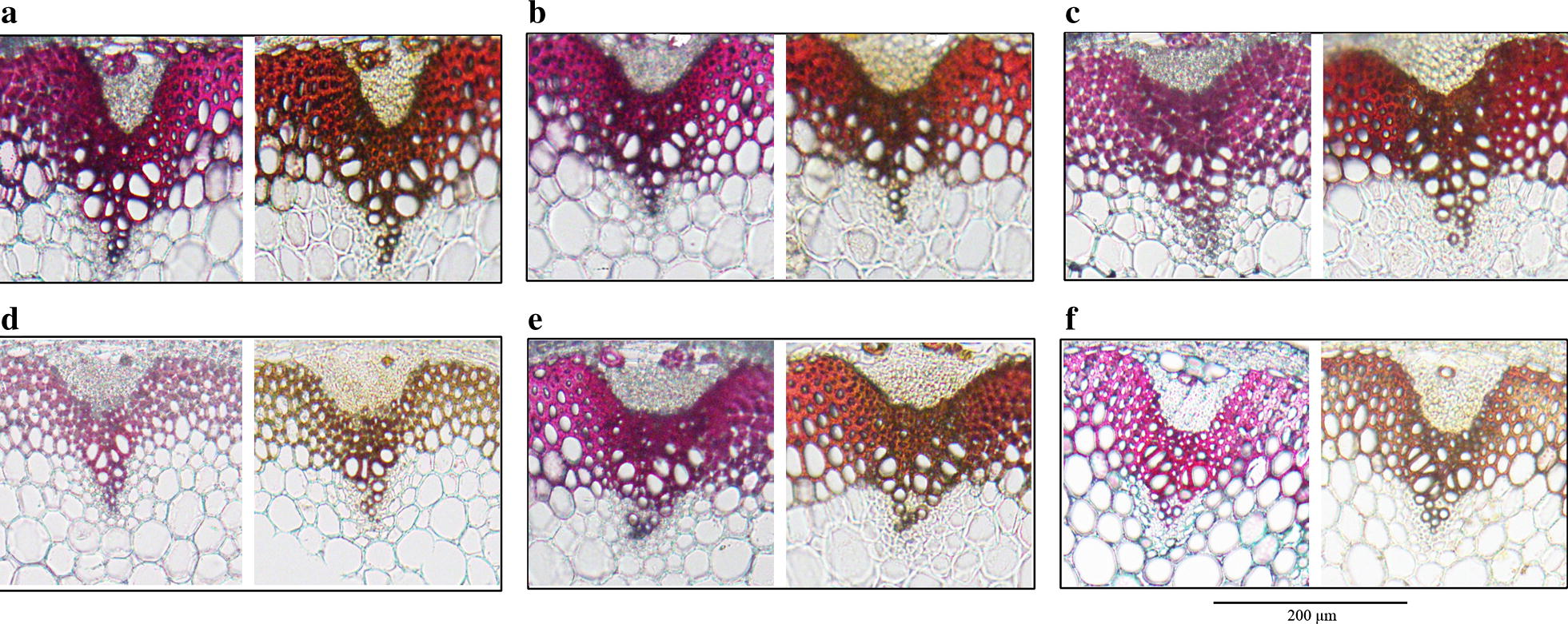
Stem cross section of representative *A. thaliana* lines stained by Wiesner (left) and Maule (right) reactions. **a** WT; **b** *WT*-*35S:NcCSE*; **c** *WT*-*35S:NcHCT*; **d** *cse*; **e** *cse*-*35S:NcCSE*; **f** c*se*-*35S:NcHCT*; bar = 200 µm

The lignin content always changes along with CWR (Shinya et al. 2016). To observe the difference in content between the different lines microscopically, safranin and fast green staining as described in “Materials and methods” section was used. Comparing the staining of the *cse, cse*-*35S:NcCSE* and *cse*-*35S:NcHCT* lines, the xylem of the *cse* mutant was almost blue, while that of the *cse*-*35S::NcCSE* was red, and the color of *cse*-*35S::NcHCT* xylem was intermediate between blue and red. WT was stained a red color (Fig. 4). Furthermore, when Wiesner’s phloroglucinol-based staining method was applied to representative lines, including the *cse* mutant, *cse*-*35S::NcHCT* lines, *cse*-*35S::NcCSE* lines and WT, the xylem was stained in colors ranging from light red in the *cse* mutant to dark red in WT. The color of *cse*-*35S::NcHCT* and *cse*-*35S::NcCSE* lines was intermediate, but *cse*-*35S::NcCSE* lines were much darker in color than *cse*-*35S:NcHCT* lines. When stained with Maule’s reagent, the xylem of the *cse* mutant was brown while that of WT was stained red; the color of the xylem in *cse*-*35S::NcCSE* lines was similar to but not identical with that of WT, but the color of the *cse*-*35S::NcHCT* lines was an orange shade in between red and brown (Fig. 5).

### Lignin content analysis

In this study, the lignin content was reduced by 23.3% and 31.7% in, respectively, the stems and leaves of the *cse* mutant compared with WT (Additional file 1: Table S8). This severe deficiency was completely complemented by *NcCSE* overexpression. For instance, the lignin content of both stems and leaves of the three *cse*-*35S::NcCSE* lines was not significantly different from WT levels. Although all three *cse*-*35S::NcHCT* lines had significantly more lignin than the *cse* mutant, except that the stem lignin content of *cse*-*35S::NcHCT2* was at the same level as that in *cse*, the lignin contents of these line, were not restored to WT level, apart from the stem lignin content of *cse*-*35S::NcHCT1* which was not significantly less than WT (Additional file 1: Table S8).

### Lignin monomer composition analysis

The *cse* mutant was found to have a dramatic increase in H monomer but a decrease in G monomer and S monomer in the stem. This change in lignin monomer composition resulted in the S/G ratio increasing from 0.44 (WT) to 0.84 (*cse*). The S/G ratio (0.57) was partially restored to, but was still significantly higher than, the WT level in *cse*-*35S::NcCSE* lines. In *cse*-*35S::NcHCT* lines, both the decrease in H monomer content and the increase in G monomer content were at a significant level compared with *cse*, but S monomer content showed no significant change, so the S/G ratio was intermediate between that of *cse* mutant and that of the *cse*-*35S::NcCSE* line (Table 2, Additional file 1: Fig. S4).

**Table 2:** Lignin monomer composition of representative lines

Line	H/CWR (μmol/g)	G/CWR (μmol/g)	S/CWR (μmol/g)	S/G
WT	0.37 (0.1)	62.3 (2.1)	27.6 (1.2)	0.443018
cse	6.9 (0.5)***	20.9 (0.4)***	17.6 (0.9)***	0.842105***
*cse*-*35S::NcCSE*	0.83 (0.2)**^†††^	53.2 (2.6)**^†††^	30.4 (2.2)*^†††^	0.571429**^†††^
*cse*-*35S::NcHCT*	5.7 (0.2)***^†^	23.7 (0.5)***^†^	18.5 (0.7)***	0.780591***

The data outside and inside the brackets are mean value and SEM (n = 3)

* Significant difference compared with WT, ^†^indicates significant difference compared with *cse* (* and ^†^ 0.01 < p<0.05), (** and ^††^ 0.001 < p < 0.01), (*** and ^†††^ p < 0.001)

## Discussion

### CSE is a key enzyme in lignin biosynthesis

The lignin biosynthetic pathway has been well known for years, but it was not found that CSE, together with 4CL, bypassed the second HCT reaction, or that its preferred substrate was caffeoyl shikimate, until 2013 (Vanholme et al. 2013). The *cse* mutation was shown to reduce the expression levels of lignin biosynthesis genes upstream of it, and so block the lignin biosynthetic pathway (Vargas et al. 2016). Similarly, in this study, the lignin biosynthesis genes upstream of *AtCSE*, including *C3H*, *C4H*, *HCT*, were down regulated, but the gene *CCoAOMT1*, which lies downstream of *CSE* in the pathway, was not, the inflorescence stems were smaller and lighter, the CWR and lignin content were reduced in both the leaves and the stem, and the S/G ratio increased dramatically in the *cse* mutant compared with WT. However, the increases in expression levels of *C3H*, *C4H*, *HCT* and *CCoAOMT1* (Fig. 1), and the restoration of lignin deposition (Additional file 1: Table S8) in *cse*-*35S::NcCSE* lines were also accompanied by restored plant growth (Fig. 3a, d). The mutant phenotype of *cse* was thus complemented in stable transgenic lines expressing *NcCSE.* All of these findings indicate that the *N. cadamba* gene *NcCSE* has a similar function to *AtCSE* in *A. thaliana*.

### CSE affects lignin monomer content

In this study, the *cse* mutant was found to have a much higher content of H monomer and lower content of G and S monomer in the stem compared with WT, resulting in the S/G ratio increasing from 0.44 (WT) to 0.84 (*cse*). This is likely to be because CSE plays an important role in the part of the phenylpropanoid pathway leading to the G and S units after the branching off of H unit biosynthesis (Vanholme et al. 2013). The mutant phenotype of *cse* was greatly complemented but not completely restored to WT in the stable transgenic lines *cse*-*35S::NcCSE*, though there was still a significant difference in the amount of each lignin unit between WT and *cse*-*35S::NcCSE*. The lignin unit content of *cse* was less fully restored in the stable transgenic lines *cse*-*35S::NcHCT*, which had more H units, less of the G and S units and a higher S/G ratio compared with WT (Table 2, Fig. 5). Furthermore, lignin biosynthesis genes including *C3H*, *C4H*, *HCT*, *CCoAOMT1* were up regulated in the stable transgenic lines *cse*-*35S::NcCSE* but were not affected in the transgenic lines *cse*-*35S::NcHCT* compared with the *cse* mutant (Fig. 1), and alterations in the activity of these related enzymes is also likely to have affected the composition of lignin units (Franke et al. 2002; Besseau et al. 2007; Li et al. 2010). These findings again suggest that *NcCSE* has a similar function to *AtCSE* and also that it is more effective than *NcHCT* in restoring the distribution of lignin units content in *A. thaliana*, and that *NcCSE* may be more active than *NcHCT* in the section of the *N. cadamba* phenylpropanoid pathway that leads to to G and S units after the branching off of of H unit biosynthesis.

### NcCSE may be more active than NcHCT

In the study, the mutant phenotypes of *cse*-*2* were complemented, either completely or partially, in the stable transgenic lines *cse*-*35S::NcCSE*; these characters included height and dry weight of the main inflorescence stem, dry weight of the total aboveground part of the plant, and CWR and lignin content of stems and rosette leaves. However, the phenotypes of the stable transgenic lines *cse*-*35S::NcHCT* were not restored to WT, in comparison to which they showed significant differences (Additional file 1: Tables S7, S8). Additionally, the xylem of *cse*-*35S::NcCSE* and WT was red, but the color of *cse*-*35S::NcHCT* xylem lay in between blue and red, in the safranin and fast green staining experiment (Fig. 4). When stained with phloroglucinol, the xylem of the *cse* mutant was light red but the the xylem of WT was dark red. The color of *cse*-*35S:NcHCT* and *cse*-*35S:NcCSE* lines was intermediate, but *cse*-*35S:NcCSE* lines were much darker in color compared with *cse*-*35S:NcHCT* lines (Fig. 5). All of these observations indicate that the lignin content was higher in the *cse*-*35S::NcCSE* lines compared with the *cse*-*35S::NcHCT* lines. When lignin content was measured, the results showed that it was reduced by 23.3% in the stem of the *cse* mutant compared with WT and this severe deficiency was completely complemented by *NcCSE* overexpression, but *cse*-*35S::NcHCT* lines did not show full complementation, with the exception of *cse*-*35S::NcHCT1* (Additional file 1: Table S8). CSE and HCT have the same substrate, caffeoyl shikimate (Vanholme et al. 2013). Both *NcCSE* and *NcHCT* were expressed at the highest level in the stem segment of *N. cadamba* with the greatest degree of lignification; however, the expression level of *NcCSE* was much higher than that of *NcHCT* in the same segment (Table 1) (Ouyang et al. 2016). These results suggest that NcCSE may be more active than NcHCT in lignin biosynthesis in *N. cadamba*.

### Potential application of *NcCSE* down-regulation

Lignin does not reduce the palatability of forage grass for animals (Cornelissen et al. 2014), but it hinders the isolation of cellulose fibers and the efficient enzymatic depolymerization of cellulose and hemicellulose into fermentable sugars by limiting the access of the hydrolytic enzymes to their polysaccharide substrate in the biorefining industry (Vanholme et al. 2013). In a previous study, silencing CSE in poplar did not drastically affect plant growth or development, but it reduced lignin deposition and flux into G and S units, increased the cellulose content and improved the saccharification efficiency for stems (Saleme et al. 2017). We found that the function of *NcCSE* was similar to that of *AtCSE* in lignin biosynthesis. A highly efficient in vitro regeneration system has been successfully established for *N. cadamba* (Huang et al. 2014), and in recent years the CRISPR–Cas9 system for genome editing has been established and applied widely to create a loss-of-function mutants affecting of specific genes (Mali et al. 2013; Hsu et al. 2014). These factors suggest that the lignin content in both stems and leaves of *N. cadamba* could be reduced by creating the loss-of-function mutant *nccse* with the CRISPR–Cas9 system, resulting in improvements in the nutrient absorption of the leaves for animals and in the total saccharification yield from the stems.

## Supplementary information

**Additional file 1.** Additional tables and figures.

## Data Availability

All the gene sequence is deposited into NCBI, all the vectors and materials are stored in our lab, all the data are either in the manuscript or the additional data.
