# Supplementary material for: Cloning and characterization of the lignin biosynthesis genes NcCSE and NcHCT from Neolamarckia cadamba
Source: AMB Express. 2019 Sep 21;9:152. doi: 10.1186/s13568-019-0860-z (PMC6754823; doi:10.1186/s13568-019-0860-z)
Supplement: Supplementary file 1 — Additional file 1. Additional tables and figures. [file 13568_2019_860_MOESM1_ESM.zip › Suppliment data.docx]

**Cloning and characterization of the lignin biosynthesis genes *NcCSE* and *NcHCT* from** ***Neolamarckia cadamba***

Juncheng Li^1,2,3,4&^, Xiaoling Huang^1&^, Hao Huang^5^, Heqiang Huo^6^, Chi D Nguyen^6^, Ruiqi Pian^1,3^, Huaqiang Li^7^,Kunxi Ouyang^1,3#^, Xiaoyang Chen^1,3,4#^

1. Guangdong Key laboratory for innovative Development and Utilization of ForestPlant Germplasm, South China Agricultural University, Wushan Road 483, Tianhe District, Guangzhou 510642, China
2. Guangdong Key Laboratory of Tropical and Subtropical Fruit Tree Research, Institute of Fruit Tree Research, Guangdong Academy of Agricultural Sciences, Guangzhou 510640, China
3. Guangdong Province Research Center of woody forage engineering technology, College of Forestry and Landscape Architecture, South China Agricultural University, Guangzhou, 510642, China
4. State Key Laboratory for Conservaion and Utilization of Subtropical Agro-bioresources
5. Guangxi Botanical Garden of Medical Plants, Nanning 530023, China
6. Mid-Florida Research and Education Center, Institute of Food and Agricultural Sciences, University of Florida, Apopka, FL, USA
7. State_run Leizhou Forestry Bureau,Zhanjiang 524348,Guangdong,China

Corresponding author:

Xiaoyang Chen

E-mail: chen_author@qq.com, Telephone:18814113792, Fax:020-85280001

Kunxi Ouyang

E-mail: kunxi_123@163.com, Telephone:15018746300, Fax: 020-85280001;

&:these authors contributed equally to this study

#: these corresponding authors contributed equally to this study

Table S1. Nutrition of the leaves of *N.Cadamba* and *Medicago sativa*

| Items | *N.Cadamba* | *Medicago sativa* |
| --- | --- | --- |
| Crude protein% | 22.89 | 16～20 |
| Crude fat% | 3.86 | 1.53 |
| Crude fiber% | 11.91 | 36.07 |
| Crude ash% | 7.87 | 10～12 |
| Total energy(MJ kg^-1^) | 18.1 | 17.37 |

Table S2. Primer list for clone *NcCSE* and *NcHCT*

| Primer | Sequence |
| --- | --- |
| NcCSE-N1-F | CTCAAGACTCCCAAACTCC |
| NcCSE-N1-R | GCACTGACAAACGCAAT |
| NcCSE-BP-F | ggggacaagtttgtacaaaaaagcaggcttcATGGCAAGCGAAATTGACAA |
| NcCSE-BP-R | ggggaccactttgtacaagaaagctgggtgTCACTGGCTTGTGCCTTCTT |
| NcHCT-N1-F | CCCATAATACTCTTCTTCTCAA |
| NcHCT-N1-R | GAAACAACCACCACATCAA |
| NcHCT-BP-F | ggggacaagtttgtacaaaaaagcaggcttcATGAAAATCGAGGTTAGAGATTCGA |
| NcHCT-BP-R | ggggaccactttgtacaagaaagctgggtgTAAACCTTCATATGCAATACCACCA |

The lower letter in the sequence is BP adapter.

Table S3. Primer list for RT-PCR, *Cyclophilin* and *PP2AA3*are reference genes for N.Cadamba and A.thaliana

| Gene ID | Symbol | Forward primers | Reverse primers |
| --- | --- | --- | --- |
| JX902587 | *Cyclophilin* | GACAGGAGGAGAATCTATCTATGG | AACCTGCCCAAACACCACAT |
| MG739672 | *NcCSE* | CAAGATGTTATTGACGCAGCC | ATCGGTCACTTTGTCATCTCC |
| MG739673 | *NcHCT* | AGGTCTGCTCTTGATTACTTGG | CAGGCCCCATAAATATAGGACG |
| AT1G13320 | *PP2AA3* | TAACGTGGCCAAAATGATGC | GTTCTCCACAACCGCTTGGT |
| AT4G36220 | *FAH1/F5H* | AGTCATGGGCTTCAGTTCGTGATGA | AAACGCTGCCCGGTAAGTTATGTTG |
| AT4G34050 | *CCoAOMT1* | CGCCAAGAACACAATGGAGATCG | CCGGCTTTCTCAATGATCGGTAAAC |
| AT5G48930 | *HCT* | GAATTCCATACGAGGGTTTGTCTT | GGGCAATGGCAACGGATA |
| AT2G30490 | *C4H* | CACCGGGAAAGGTCAAGATA | CCCAACCTTCACGATTCTGT |
| AT2G40890 | *C3H* | TTTCGTTGATGCGTTGCTAA | CGGTCAAGTCCAACCACTCT |
| AT1G52760 | *CSE* | CTCTTTGGTTTGGCTGATACG | CAGTAACTCTCTCATTGTTCCCAC |
| AT2G37090 | *IRX9* | GAAGGGCCTGTTTGTGAATC | TTATCGAATCCTGTTTGGTGC |
| AT5G67210 | *IRX15-L* | ACCCTTCTCTGGAAATCGCT | GAAGACTTCGATCTCCGGGT |
| AT5G67220 | *AT5G67220* | CACCACCTGTAAGGAGGACA | TGCACCATAATTTCCTCGCC |
| AT2G03200 | *AT2G03200* | TTGGATCTTTAGCCTCGGGT | GAAAGGCGTTTTGCTCCAAC |
| AT2G45290 | *TKL2* | AGCCACTACTGGTCCTCTTG | GTCCACGCTCTCGGTAAAAG |
| AT3G23090 | *WDL3* | CTAGTGAGATCCCCGTTGGT | CCTAGGTACCTTTGTTGCCTG |
| AT5G01360 | *TBL3* | CATACTAGAAGTGCGGATTGGG | GAGAGCTGCGTGATGTTGAT |
| AT2G40120 | *AT2G40120* | CTGGAAAAACAGGACGGGATT | GCCTGAACCACCTTGCTAAA |
| AT5G42180 | *PER64* | GTTGCTCTCTCTGGAGGTCA | TTCTTCACCGTGTTATGGGC |
| AT1G72230 | *AT1G72230* | GCGATACCATCGTGTTCAACT | TTCCGTCTTGTACGGGTGTA |
| AT5G40020 | *AT5G40020* | TCTGCCATGCGGAGGTATTA | GATGTCCATCACAATCACCTGT |
| AT1G73640 | *RABA6a* | GACGAGTTCCGGTTTGACTC | TTGCTCGAAATCTTTCTTGGC |
| AT1G23260 | *MMZ1* | CAGACCCGGATAAACATGGC | AGCTTCCTCGTTACCTTCCG |
| AT5G24310 | *ABIL3* | GTTGCTATCCGACCGTCATC | GGTTCTGACGGATACCGTTT |
| AT1G33800 | *ATGXMT1* | GGTGGTCGTACCTTGTTTATTG | GTAAAAGTCCGCCGGGAAAT |
| AT5G54690 | *IRX8* | CTCGAATCCGACAATTGCCAAAAAC | GCATATGCCAGAACGGATCTATGGT |
| AT1G27440 | *IRX10* | ACCCCGAAGGTGGATATTATGCAAG | AACTGGAATGCACCCAAACACAAC |
| AT4G32410 | *CESA1* | GATGGACCCGGCTATTGGAAAGAAG | TACTGGACCCTGGATACCATCCAAC |
| AT5G44030 | *CESA4* | GATGCGGAGTGGAAAGAACGTGT | TTGGCTGGTGTCAAGATACGGAAAC |
| AT5G17420 | *CESA7* | TGCCAACACAACAATCTACCCCTTC | CCGCTCCATCTCAATTCCAAGATTC |
| AT4G18780 | *CESA8* | CATGAGGCTCAGATTCCTACCCAAC | CTATCGACAGGATGCGTGATACGG |

Table S4. Chi-Square Goodness-of-Fit Test of transgenic lines as single copy insertion

| Lines | Etiolated  seedlings | Numbers in theory | Normal seedlings | Number in theory | χ_c_^2^ | χ^2^_0.05(1）_ |
| --- | --- | --- | --- | --- | --- | --- |
| *cse-35S:NcCSE-1* | 30 | 24 | 66 | 72 | 1.68 | 3.84 |
| *cse-35S:NcCSE-2* | 26 | 23.25 | 67 | 69.75 | 0.29 | 3.84 |
| *cse-35S:NcCSE-3* | 32 | 28 | 80 | 84 | 0.58 | 3.84 |
| *cse-35S:NcHCT-1* | 23 | 25.5 | 79 | 76.5 | 0.21 | 3.84 |
| *cse-35S:NcHCT-2* | 26 | 22.5 | 64 | 67.5 | 0.53 | 3.84 |
| *cse-35S:NcHCT-3* | 28 | 23 | 64 | 69 | 1.17 | 3.84 |
| *WT-35S：NcCSE-1* | 26 | 23.75 | 69 | 71.25 | 0.17 | 3.84 |
| *WT-35S：NcCSE-2* | 28 | 23.25 | 65 | 69.75 | 1.04 | 3.84 |
| *WT-35S：NcCSE-3* | 21 | 26.5 | 85 | 79.5 | 1.26 | 3.84 |
| *WT-35S:NcHCT-1* | 28 | 24 | 68 | 72 | 0.68 | 3.84 |
| *WT-35S:NcHCT-2* | 31 | 25 | 69 | 75 | 1.61 | 3.84 |
| *WT-35S:NcHCT-3* | 27 | 24.75 | 72 | 74.25 | 0.16 | 3.84 |

Table S5. Chi-Square Goodness-of-Fit Test of transgenic lines as double copy insertions

| Lines | Etiolated  seedlings | Numbers in theory | Normal seedlings | Number in theory | χ_c_^2^ | χ^2^_0.001(1）_ |
| --- | --- | --- | --- | --- | --- | --- |
| *cse-35S:NcCSE-1* | 30 | 6 | 66 | 90 | 98.18 | 10.83 |
| *cse-35S:NcCSE-2* | 26 | 5.81 | 67 | 87.19 | 71.13 | 10.83 |
| *cse-35S:NcCSE-3* | 32 | 7 | 80 | 105 | 91.47 | 10.83 |
| *cse-35S:NcHCT-1* | 23 | 6.38 | 79 | 95.63 | 43.51 | 10.83 |
| *cse-35S:NcHCT-2* | 26 | 5.63 | 64 | 84.38 | 74.91 | 10.83 |
| *cse-35S:NcHCT-3* | 28 | 5.75 | 64 | 86.25 | 87.76 | 10.83 |
| *WT-35S-NcCSE-1* | 26 | 5.94 | 69 | 89.06 | 68.75 | 10.83 |
| *WT-35S-NcCSE-2* | 28 | 5.81 | 65 | 87.19 | 86.31 | 10.83 |
| *WT-35S-NcCSE-3* | 21 | 6.63 | 85 | 99.38 | 31 | 10.83 |
| *WT-35S-NcHCT-1* | 28 | 6 | 68 | 90 | 82.18 | 10.83 |
| *WT-35S-NcHCT-2* | 31 | 6.25 | 69 | 93.75 | 100.36 | 10.83 |
| *WT-35S-NcHCT-3* | 27 | 6.19 | 72 | 92.81 | 71.13 | 10.83 |

Table S7. Average plant height, stem diameter and hypocotyls length at 7^th^ and 14^th^ day of the 14 lines.

| Lines | Height (cm) | Stem diameter(mm^2^) | 7th day hypocotyls length | 14th day hypocotyls length |
| --- | --- | --- | --- | --- |
| WT | 38.9(0.63) | 1067.8(17.87) | 3.89(0.047) | 4.48(0.043) |
| *cse* | 25.6(0.25)*** | 1015.1(14.77)* | 3.87(0.034) | 4.49(0.026) |
| *cse-35S：NcCSE1* | 38(0.87) ††† | 1088.7(14.67) ††† | 3.9(0.034) | 4.48(0.083) |
| *cse-35S：NcCSE2* | 39.3(0.97) ††† | 1060.5(24.46) | 3.93(0.036) | 4.43(0.065) |
| *cse-35S：NcCSE3* | 38.6(0.54) ††† | 1076.8(20.98) † | 3.88(0.041) | 4.44(0.072) |
| *cse-35S：NcHCT1* | 28.5(0.47)***††† | 1066.8(15.07) † | 3.9(0.043) | 4.54(0.057) |
| *cse-35S：NcHCT2* | 28.5(0.52)***††† | 1073.8(17.49) † | 3.91(0.03) | 4.43(0.064) |
| *cse-35S：NcHCT3* | 27.8(0.76)***†† | 1041.3(16.62) | 4(0.039) | 4.53(0.054) |
| *WT-35S:NcCSE1* | 38.8(0.62) ††† | 1039.5(24.78) | 3.92(0.039) | 4.51(0.034) |
| *WT-35S:NcCSE2* | 39.3(0.87) ††† | 1045.8(21.54) | 3.92(0.045) | 4.44(0.069) |
| *WT-35S:NcCSE3* | 38.6(1.03) ††† | 1071(25.86) | 3.9(0.035) | 4.43(0.059) |
| *WT-35S:NcHCT1* | 40.3(1.05) ††† | 1102.6(15.6) ††† | 3.97(0.043) | 4.43(0.06) |
| *WT-35S:NcHCT2* | 40.3(1.1) ††† | 1042.6(22.02) | 3.91(0.037) | 4.49(0.057) |
| *WT-35S:NcHCT3* | 40.2(0.74) ††† | 1080.4(15.41) †† | 3.9(0.044) | 4.52(0.068) |

The data outside and inside the brackets are mean value and SEM (n=30), * means significant difference compare with WT, † means significant difference compare with *cse*, * and † means 0.01<p<0.05, ** and †† means 0.001<p<0.01, *** and ††† means p<0.0001.

Table S8. Comparison of some secondary growth characters between different transgenic and wild type lines.

| Lines | Stem | | | |  | Rosette Leaves | | | | | |  | Whole Plant |
| --- | --- | --- | --- | --- | --- | --- | --- | --- | --- | --- | --- | --- | --- |
|  | Dry Weight(mg) | CWR(%DW) | Lignin(%CWR) | Cellulose(%CWR) |  | Dry Weight(mg) | CWR(%DW) | Lignin(%CWR) | Cellulose(%CWR) | Leaf Area(mm^2^) | Leaves Numbers |  | Dry Weight(mg) |
| WT | 73.3(1.6) | 79.1(0.9) | 16.67(0.58) | 54.25(1.24) |  | 122.3(3.53) | 22.45(0.54) | 14.9(0.36) | 42.74(0.3) | 88.14(2.53) | 10(0.42) |  | 956.3(18.99) |
| *cse* | 39.4(1.24) | 70.8(0.94)*** | 12.78(0.41)*** | 43.12(0.78)*** |  | 115.4(2.81) | 19.24(0.34)*** | 10.18(0.32)*** | 31.48(0.37)*** | 89.13(2.16) | 11.6(0.45)* |  | 925.3(20.09) |
| *cse-35S：NcCSE1* | 66.7(1.62)** | 76.7(0.22)*††† | 15.43(0.5) †† | 53.11(1.87) †† |  | 131.2(3.13) ††† | 20.51(0.6)*† | 14.44(0.42) ††† | 44.06(0.2) ††† | 81.46(1.54)*†† | 10.1(0.38) † |  | 948.8(18.61) |
| *cse-35S：NcCSE2* | 74.3(1.59) ††† | 74.6(0.63)***†† | 17.11(0.56) ††† | 53.18(1.24) ††† |  | 129(3.96) †† | 20.71(0.6)*† | 15.7(0.24) ††† | 44.4(1.35) ††† | 81.61(0.74)*†† | 10.4(0.34) † |  | 954(17.75) |
| *cse-35S：NcCSE3* | 69(0.78)*††† | 78.6(0.75) ††† | 17.52(0.51) ††† | 56.55(2.23) ††† |  | 126.3(3.7) † | 21.7(0.56) †† | 14.77(0.26) ††† | 42.9(0.82) ††† | 84.01(2.95) | 10.3(0.47) |  | 947.6(19.73) |
| *cse-35S：NcHCT1* | 59.9(1.26)***††† | 71.2(0.53)*** | 15.14(0.52) †† | 46.61(0.44)***†† |  | 126(3.69) † | 20.89(1.11) | 13(0.54)*†† | 40.55(1.24)*†† | 85.77(0.37) | 9.9(0.31) †† |  | 949.9(13.16) |
| *cse-35S：NcHCT2* | 60.7(1.94)***††† | 71.4(1.1)*** | 12.9(0.38)*** | 49.97(1.08)*††† |  | 128.4(4.94) † | 19.61(0.52)** | 11.96(0.17)***†† | 39.8(0.52)***†† | 88.95(1.53) | 9.8(0.36) †† |  | 981.6(19.58) |
| *cse-35S：NcHCT3* | 66.3(2.28)*††† | 68.8(0.45)*** | 14.22(0.38)**† | 48.31(1.14)**†† |  | 117(3.49) | 20.01(0.49)** | 12.77(0.36)**††† | 39.12(1.01)**††† | 86(2.11) | 10.2(0.29) † |  | 956.3(18.25) |
| *WT-35S:NcCSE1* | 71(1.72) ††† | 79.1(0.51) ††† | 17.18(0.32) ††† | 55.76(1.09) ††† |  | 122.6(4.53) | 21.79(0.88) † | 15.09(0.41) ††† | 41.28(1.12) ††† | 93.22(3.94) | 10.2(0.33) † |  | 969.2(22.49) |
| *WT-35S:NcCSE2* | 73.8(2.61) ††† | 79.4(0.45) ††† | 17.94(0.47) ††† | 53.78(1.88) ††† |  | 133.2(4.14) ††† | 23.38(0.74) ††† | 13.98(0.75) †† | 46.37(1.03) †† | 87.77(1.13) | 9.9(0.31) †† |  | 921.1(19.05) |
| *WT-35S:NcCSE3* | 66.8(2.71) ††† | 78(0.69) ††† | 15.59(0.64) †† | 52.98(1.65) ††† |  | 121.5(3.36) | 21.66(0.44) ††† | 13.97(0.3) ††† | 42.82(1.64) ††† | 91.03(2.22) | 10.2(0.33) † |  | 966.5(20.98) |
| *WT-35S:NcHCT1* | 68.8(1.8) ††† | 77.3(0.34) ††† | 17.75(0.57) ††† | 48.72(1.21)*†† |  | 131.6(3.18) ††† | 23.21(0.6) ††† | 15.64(0.53) ††† | 44.82(1.2) ††† | 84.11(1.3) | 10.3(0.37) † |  | 941.3(18.95) |
| *WT-35S:NcHCT2* | 71.5(1.68) ††† | 77(0.64) ††† | 15.03(0.3)*†† | 50.03(1.85) †† |  | 128.7(4.3) † | 23.5(0.26) ††† | 14.98(0.49) ††† | 43.48(0.99) ††† | 89.49(4.48) | 9.9(0.43) † |  | 971.1(20.99) |
| *WT-35S:NcHCT3* | 75.2(2.41) ††† | 78.2(0.43) ††† | 16.32(0.32) ††† | 58.99(0.96)*††† |  | 133(4.79) †† | 21.53(0.64) †† | 15.02(0.42) ††† | 45.13(1.7) ††† | 88.52(1.49) | 10.1(0.31) † |  | 950.9(22.69) |

The data outside and inside the brackets are mean value and SEM (n=5), * means significant difference compare with WT, † means significant difference compare with *cse*, * and † means 0.01<p<0.05, ** and †† means 0.001<p<0.01, *** and ††† means p<0.0001.


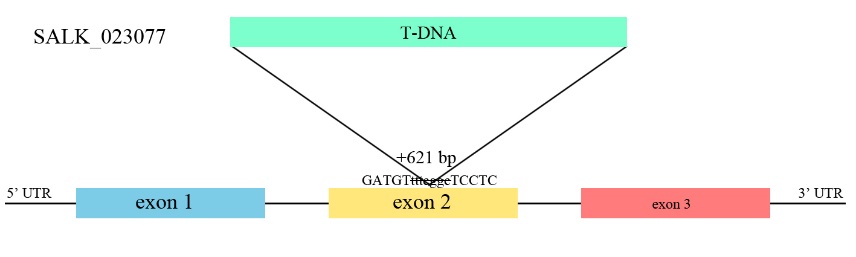


Figure S1. T-DNA insertion position of *cse* mutant (SALK_023077).


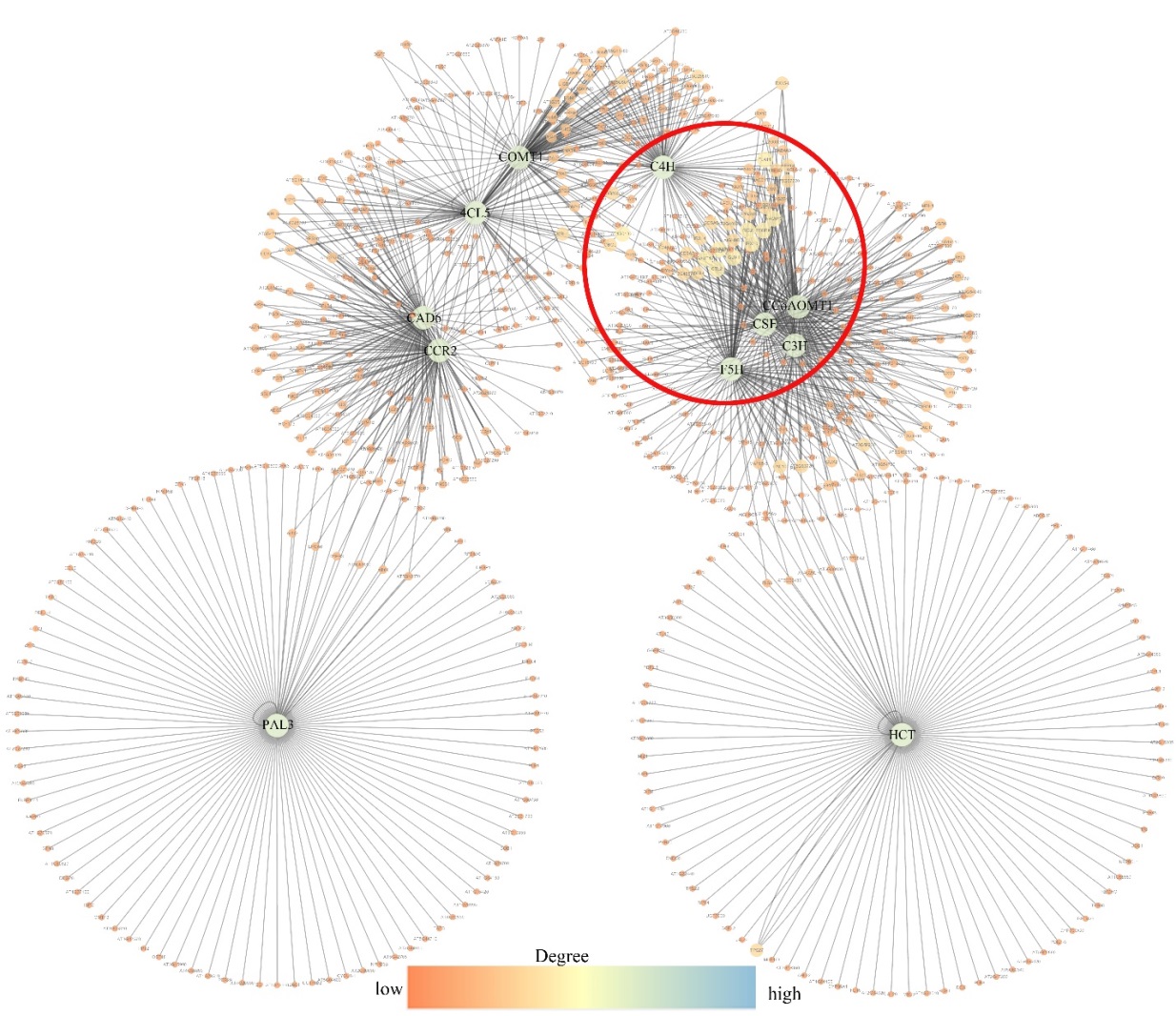


Figure S2. Genes co-expression with the 11 lignin biosynthesis pathway genes

First 100 genes that co-expressed with those lignin biosynthesis pathway were showed in this figure, those genes have some shared co-expression genes, the part inside the red box shows that*CSE* shared more co-expression genes with *F5H*, *C3H*, *C4H* and *CCoAOMT1* compare other lignin biosynthesis pathway genes.


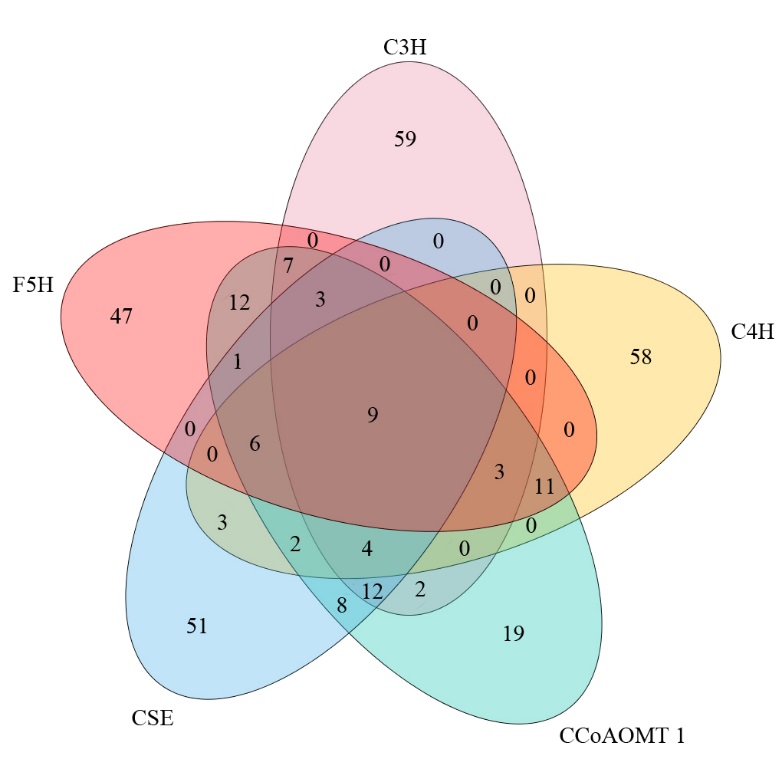


Figure S3. Venn diagram illustrating shows the numbers of genes shared with each other between*CSE, F5H*, *C3H*, *C4H* and *CCoAOMT1*.


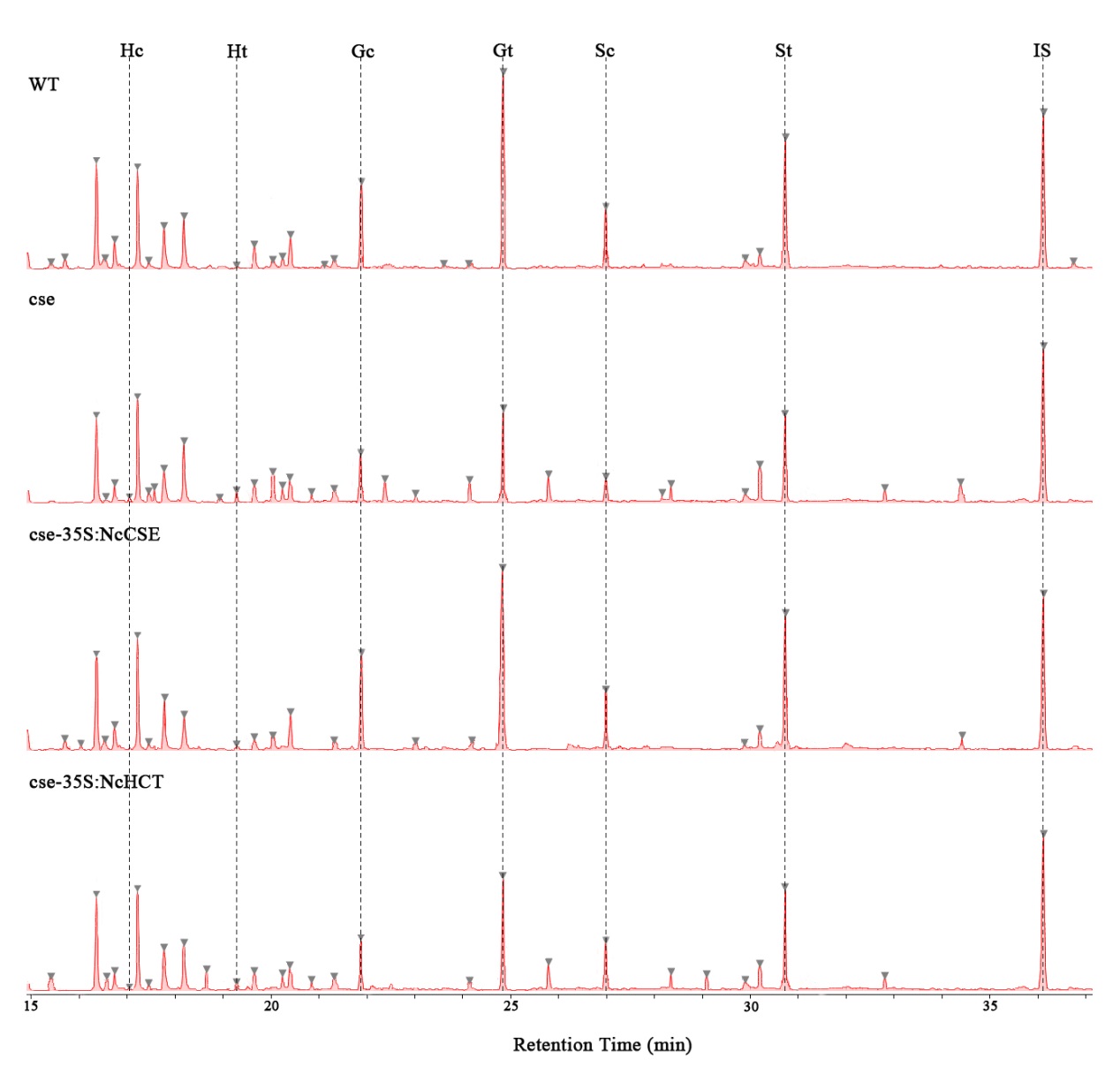


Fig. S4. GC-FID chromatograms of DFRC monomers from transgenic *A. thaliana* stem samples

H, H lignin; G, G lignin; S, S lignin; IS, internal standard (tetracosane); t, trans; c, cis.
